# Supplementary material for: Determinants Influenced by COVID-19 Vaccine: Employing the Health Action Process Approach and the Belief in Conspiracy Theories
Source: Vaccines (Basel). 2023 Mar 25;11(4):730. doi: 10.3390/vaccines11040730 (PMC10142525; doi:10.3390/vaccines11040730)
Supplement: Supplementary file 1 [file vaccines-11-00730-s001.zip › vaccines-2248781-supplementary.pdf]

Determinants Influenced by COVID-19 Vaccine: Employing the Health Action Process Approach and the Belief in Conspiracy Theories

Table S1. Items used in the study.

Factor	Code	Items	References
COVID-19 thread	PT1	I have no competence to use healthcare technology during COVID-19 pandemic.	19,20,21
	PT2	Experienced loved ones dying from COVID-19 pandemic.	
	PT3	I do not feel comfortable using healthcare technology during COVID-19 pandemic.	
	PT4	I have no access to medical care during COVID-19 pandemic.	
Openness to experience	OE1	I am curious with COVID-19 vaccine.	22,23
	OE2	I am quickly understanding COVID-19 vaccine	
	OE3	I like to present some new ideas	
Government communi-cation	GC1	Government COVID-19 Vaccine communication strategy helps individuals get correct, consistent and timely information on the new COVID-19 vaccine	26,27
	GC2	Government COVID-19 Vaccine communication strategy helps to reduce vaccine hesitancy.	
	GC3	Government COVID-19 Vaccine communication strategy helps individuals to receive the vaccine with confidence.	
	GC4	Government COVID-19 Vaccine communication strategy helps build an enabling environment to adopt and maintain COVID-19 appropriate behaviors to reduce infection risks during vaccination processes.	
Pandemic knowledge	PK1	The COVID-19 may transmit through human-to-human interaction.	29,30,31
	PK2	The COVID-19 may also transmit through a common point of contact (door, table surface, etc.).	
	PK3	The COVID-19 may transmit through handshake and communication with the carrier of this disease.	
	PK4	The initial symptoms of COVID-19 include fever, dry cough, sneezing, body aches, and breathing distress, etc.	
	PK5	The infectious diseases may be prevented if we keep ourselves clean.	
	PK6	Disease (COVID-19) can be prevented	

		through continual handwashing.	
	PK7	The COVID-19 enters the human body through the nasal (nose) and oral (mouth) cavity as well as the eyes.	
	PK8	The COVID-19 can be prevented through social distancing.	
Descriptive norm	DN1	My family members think I should take-up COVID-19 vaccine	32,33
	DN2	I know that my family members take-up COVID-19 vaccine.	
	DN3	My relatives think that I should take-up COVID-19 vaccine.	
	DN4	I know that my relative take-up COVID-19 vaccine.	
Belief in conspiracy theory	BCT1	I believe the government keeps many important secrets about the COVID-19 vaccine from the public.	14,16,17
	BCT2	I believe the COVID-19 viruses and/or diseases have been deliberately disseminated to infect certain populations.	
	BCT3	I believe the rapid spread of COVID-19 viruses and/or diseases is the result of the deliberate, concealed efforts of some organization.	
	BCT4	I believe progress toward vaccination to cure COVID-19 is deliberately being hindered.	
	BCT5	I believe groups of scientists manipulate, fabricate, or suppress information about COVID-19 vaccine to deceive the public.	
	BCT6	I believe a lot of important information regarding vaccination is deliberately concealed from the public out of self-interest.	
	BCT7	I believe the government is involved in the murder of innocent citizens for not taking precautions against the COVID-19 pandemic and not making the vaccine widely available.	
Intention to take-up COVID-19	INT1	I have high intention to be immunized with a COVID-19 vaccine	15
	INT2	I take vaccine to protect myself from COVID-19 disease currently.	
	INT3	I would recommend the COVID-19 vaccine to my friends and colleagues to get protection from COVID-19 disease.	
	INT4	Immunizing through COVID-19 vaccine is a pleasant experience for me.	
Vaccine behavior	VB1	I feel pleasing taking-up the COVID-19 vaccine.	34
	VB2	Vaccination helped me to fight against coronavirus.	

	VB3	Vaccination can be beneficial in managing my health.	
	VB4	Vaccination provides good value for healthy life style.	
Perceived benefits	PB1	Taking-up COVID-19 vaccine has many advantages	34,35
	PB2	Taking-up COVID-19 vaccine is useful for me	
	PB3	COVID-19 vaccine provides a better outcome than traditional medication.	
Value co-creation	VC1	We interact with individuals to provide better service since taking-up COVID-19 vaccine	35,36
	VC2	We interact with individuals to design offers that meet their needs after taking-up COVID-19 vaccine	
	VC3	We co-opt individual's involvement in providing services for them after taking-up COVID-19 vaccine	
	VC4	We help individuals to get more value since taking-up COVID-19 vaccine	
Continuous vaccination	CVN1	I am likely to resume taking-up vaccine in the future	37,38
	CVN2	I intend to constantly get vaccinated in the future	
	CVN3	I intend to resume taking-up vaccine rather than discontinue the vaccine.	
